# Quercetin and metformin synergistically reverse endothelial dysfunction in the isolated aorta of streptozotocin-nicotinamide- induced diabetic rats

**DOI:** 10.1038/s41598-022-25739-5

**Published:** 2022-12-10

**Authors:** Jestin Chellian, Kit-Kay Mak, Dinesh Kumar Chellappan, Purushotham Krishnappa, Mallikarjuna Rao Pichika

**Affiliations:** 1 Department of Life Sciences, School of Pharmacy, International Medical University (IMU), Bukit Jalil, 57000 Kuala Lumpur, Malaysia; 2 School of Postgraduate Studies and Research, International Medical University, Kuala Lumpur, Malaysia; 3 Department of Pharmaceutical Chemistry, School of Pharmacy, International Medical University, Kuala Lumpur, Malaysia; 4 Division of Pathology, School of Medicine, International Medical University, Kuala Lumpur, Malaysia; 5 Centre for Bioactive Molecules and Drug Delivery, Institute for Research, Development and Innovation (IRDI), International Medical University, Kuala Lumpur, Malaysia

**Keywords:** Endocrine system and metabolic diseases, Cardiology

## Abstract

The antidiabetic effects of quercetin and metformin are well known. However, their synergistic effect in reversing the symptoms of diabetes-induced endothelial dysfunction remains unknown. In this study, we have investigated their synergistic effect in streptozotocin (STZ)-nicotinamide induced diabetic rats. Seventy-five rats were divided into five groups; normal control, diabetic control, treatment groups (10 mg/kg quercetin, 180 mg/kg metformin, and combined). The plasma glucose and lipid levels, liver enzymes, *ex-vivo* studies on aortic rings, histology of liver, kidney, pancreas, abdominal aorta and thoracic aorta, and immunohistochemical studies were carried out. The findings revealed that the combination of quercetin and metformin showed a greater antidiabetic effect than either drug, and rendered protection to the endothelium. The combination effectively reversed the hyperglycemia-induced endothelial dysfunction in diabetic rats. Furthermore, it also reversed the dysregulated expression of eNOS, 3-nitrotyrosine, VCAM-1, CD31 and SIRT-1. Overall, the present study's findings demonstrate that quercetin potentiates the activity of metformin to control the complications associated with diabetes.

## Introduction

The endothelium is a permeable barrier between the bloodstream and the outer vascular wall, regulating the vascular tone and structure. Endothelial dysfunction in the diabetic state is a well-established response arising from cardiovascular risk factors. There is decreased synthesis of nitric oxide in dysregulated endothelium. In addition, its availability may be reduced significantly in association with an imbalance between the relaxation and contraction factors of the endothelium.

Diabetes mellitus is a chronic metabolic disease associated with poor glycemic control, sedentary lifestyle, and obesity^1^. It is typically the consequence of insulin resistance in which the adipose or muscle cells do not respond effectively to normal insulin levels produced by β-cells of the pancreas^2^. According to the International Diabetes Federation report, about 327 million adults were affected with diabetes in 2017. The number is expected to rise to 438 million by 2045^3^. More than 90% of diabetic cases were diagnosed with type-2 diabetes mellitus (T2DM). Uncontrolled diabetes leads to macrovascular and microvascular complications^4^. The macrovascular complications include ischemic heart disease, stroke, and foot gangrene, while the microvascular include retinopathy, nephropathy, and autonomic neuropathy^4^. Vascular disease risk reduction constitutes only to a smaller proportion. However, the key parameter, namely, glycosylated haemoglobin (HbA1C) levels, are lowered. These observations indicate that other major disease markers carry significant importance, which needs to be carefully considered to control T2DM^5^. However, reducing the glycemic level remains significant in lowering chronic cardiovascular risks. The current treatment options available in the market are expensive and have numerous side effects, including hypoglycemia, abdominal discomfort, and cell death^6^. Therefore, there has been a renewed interest in searching for newer promising diabetes treatment options.

Quercetin is a phytochemical that belongs to the class of flavonoids^7^. It is rich in tea, broccoli, and onion, exhibiting antioxidant and anti-inflammatory properties^7^. There exists a large body of evidence on Quercetin on its potential in ameliorating diabetes and related symptoms. Numerous studies have reported quercetin’s potential as an antidiabetic agent that have demonstrated improved endothelial function^8^. Quercetin is the most active flavonoid that exhibits normoglycemic properties via interacting with the 11β–Hydroxysteroid dehydrogenase type 1 (11β-HSD1) enzyme^9^. Quercetin has also been proven to enhance insulin production by 44–70% in isolated rat islets by improving the islet function^10^. The suggested mechanism includes the alteration of the Ca^2+^ fluxes and cyclic nucleotide metabolism in the islet cells^10^. Quercetin also promotes the regeneration of pancreatic islets, thus enhancing insulin production^10,11^.

Metformin was originally developed from a natural toxic compound, galegine, isolated from *Galega officinalis*. There have been numerous studies that have been carried out on the molecule ever since. In addition, several studies in the early 1950s had reported on the high efficacy and safety of metformin as an anti-diabetic agent^12^. Although it effectively reduces the elevated blood glucose in T2DM patients, its exact mechanism is still unknown^13^. Metformin suppresses glucose secretion in the liver and enhances its uptake by skeletal muscle^14,15^. Metformin reduces free fatty acid (FFA) levels and thus offers cardioprotection^16,17^. It also reduces blood pressure in humans and diabetic rats^15^.

There are no reported long-term studies on the effect of quercetin along with metformin on diabetes. Quercetin and metformin individually show improvements in vascular function. Their combination is expected to possess either additive or synergistic action. In this study, we report the synergistic effect of quercetin and metformin in streptozotocin-nicotinamide induced diabetic rats.

## Results

### Effect of treatment on oral glucose tolerance and plasma glucose

**Figure 1:**
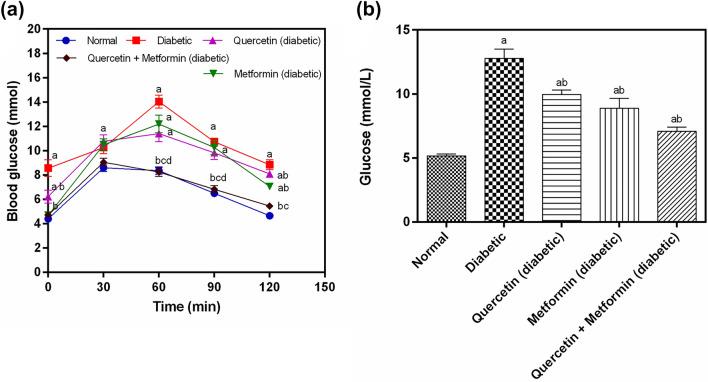
Effect of test compounds on, (**a**) oral glucose tolerance test, (**b**) on plasma glucose in diabetic rats. Data represent mean ± SEM. The alphabet, ‘a’ shows a significant difference in comparison with normal control (*p* < 0.05). The alphabet, ‘b’ shows a significant difference in comparison to diabetic control (*p* < 0.05). The alphabet, ‘c’ shows a significant difference in comparison with a quercetin treatment group (*p* < 0.05). The alphabet, ‘d’ shows a significant difference in comparison with the metformin treatment group (*p* < 0.05).

The fasting blood glucose levels were significantly (*p* < 0.05) higher in diabetic than the normal rats. The treatment with quercetin, metformin and quercetin + metformin significantly reduced the blood glucose levels in diabetic rats compared to the non-diabetic control (Fig. 1a). Among the treatment groups, rats treated with quercetin + metformin showed the highest reduction in blood glucose levels compared to quercetin and metformin alone. Besides this, quercetin + metformin-treated rats showed a glucose profile similar to the normal control (Fig. 1a).

The plasma glucose level was significantly higher (*p* < 0.05) in the diabetic control group than in the normal control. The oral treatment with quercetin, Metformin, and quercetin + Metformin for 30 days significantly reduced (*p* < 0.05) the plasma glucose level in diabetic rats compared to diabetic control. The significantly highest reduction in plasma glucose was noticed in rats treated with quercetin + metformin compared to diabetic control (Fig. 1b).

### Effect of treatment on alanine aminotransferase (ALT), aspartate aminotransferase (AST), plasma creatinine (CR) and lactate dehydrogenase (LDH)

**Figure 2:**
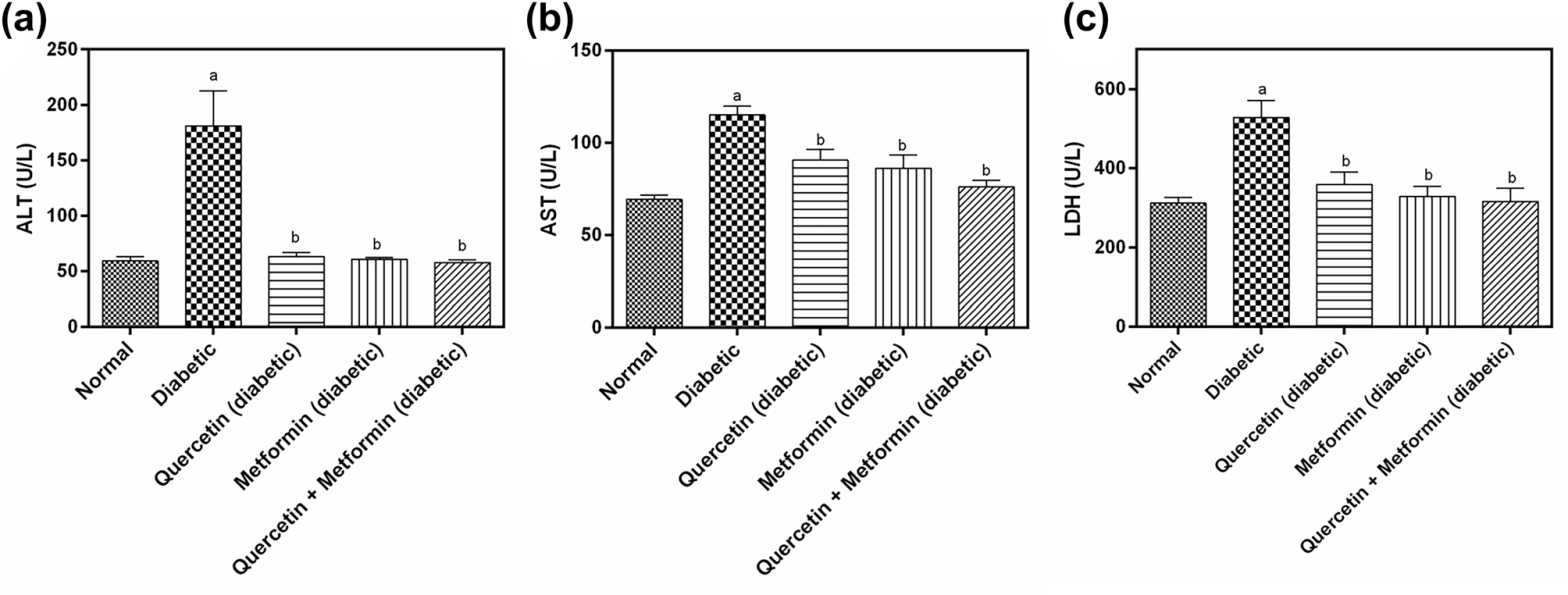
Effect of test compounds on, (**a**) plasma alanine aminotransferase (ALT), (**b**) plasma aspartate aminotransferase (AST), (**c**) plasma lactate dehydrogenase (LDH) in diabetic rats. Data represent mean ± SEM. The alphabet, ‘a’ shows a significant difference in comparison with normal control (*p* < 0.05). The alphabet, ‘b’ shows a significant difference in comparison to diabetic control (*p* < 0.05).

The plasma ALT, AST, and LDH levels were significantly higher (*p* < 0.05) in diabetic control compared with normal control. The treatment with quercetin, Metformin, and quercetin + Metformin for 30 days significantly reduced (*p* < 0.05) the plasma ALT, AST and LDH levels in diabetic rats as compared to diabetic control (Fig. 2).

### Effect of treatment on lipid profile

**Figure 3:**
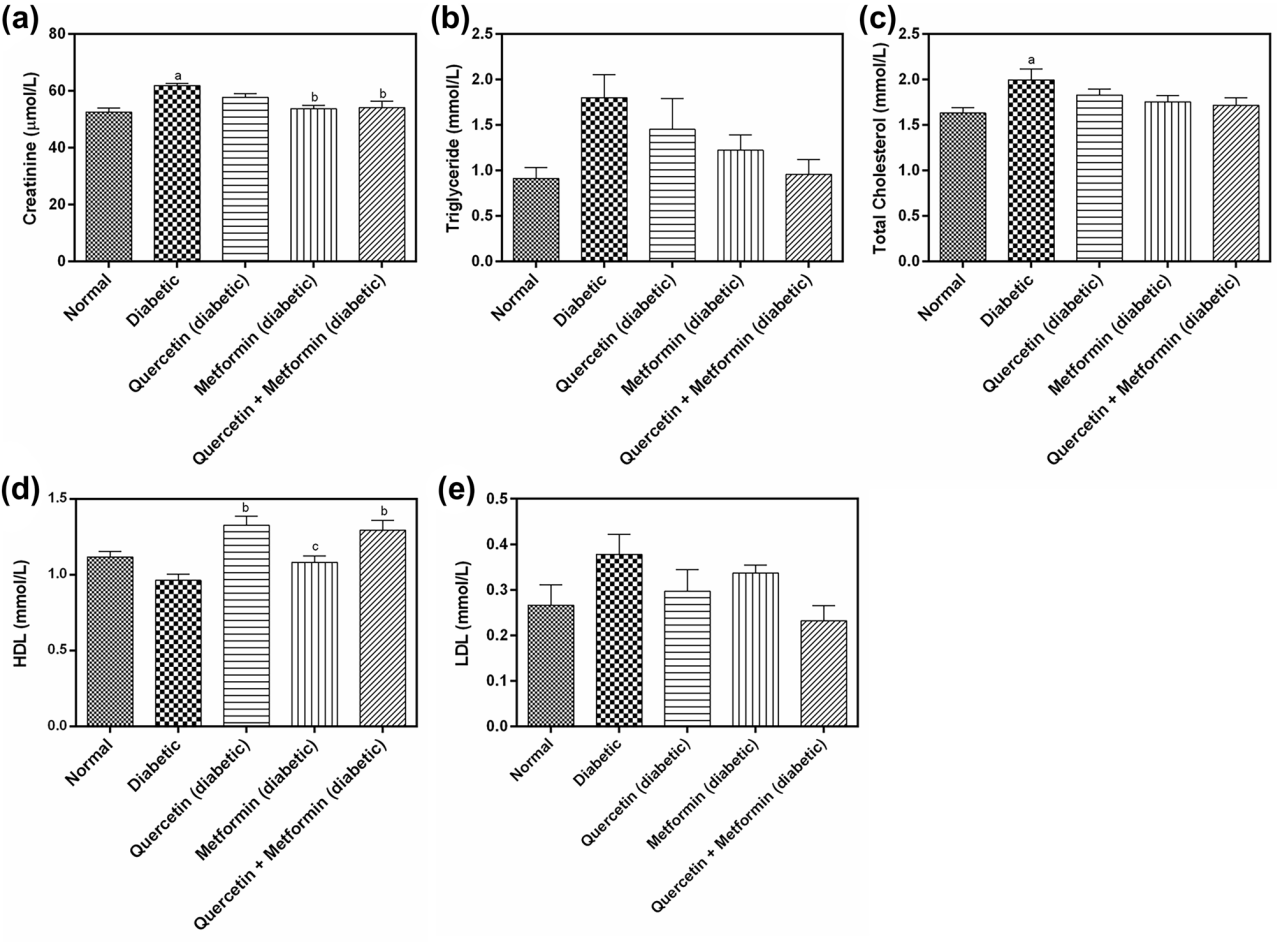
Effect of test compounds on plasma, (**a**) creatinine, (**b**) triglyceride (TG), (**c**) total cholesterol (TC), (**d**) high density lipoprotein (HDL), and (**e**) low density lipoprotein (LDL) in diabetic rats. Data represents mean ± SEM. The alphabet, ‘a’ shows a significant difference in comparison with normal control (*p* < 0.05). The alphabet, ‘b’ shows a significant difference in comparison to diabetic control (*p* < 0.05).

The mean triglyceride (TG), total cholesterol (TC), and low-density lipoprotein (LDL) levels were significantly higher (*p* < 0.05) in diabetic control as compared to normal control. In contrast, the high-density lipoprotein (HDL) level was low. Quercetin, metformin and quercetin + metformin treatments showed a reduction in CR, TG, TC and LDL levels compared to diabetic control. The quercetin + metformin was most effective among others. The HDL levels were significantly higher in quercetin, metformin, and quercetin + metformin treatment (Fig. 3).

### Ex vivo studies with rat aortic rings

Quercetin, metformin, and their combination increased the ACh-induced endothelium-dependent relaxation, SNP-induced endothelium-independent relaxation, and reduced the α 1-adrenergic agonist PE-induced contraction in diabetic rat aortic rings *ex-vivo* but had minimal effect in normal rat aortic rings (Tables 1, 2 and 3). The diabetic rats’ aorta did not relax upon the addition of acetylcholine (an endothelium-dependent vasorelaxant, Table 1), and sodium nitroprusside (an endothelium-independent vasodilator, Table 2). Phenylepinephrine (PE, α 1- adrenergic agonist) enhanced the contraction of the aorta from the diabetic rats (Table 3).

**Table 1:** Effect of test compounds on phenylephrine (PE) induced contraction in aortae.

Treatment groups	Phenylephrine (PE) induced contraction in aortae															
	Aortae with perivascular adipose tissue (PVAT)								Aortae without PVAT							
	Endothelium intact				Endothelium Denuded				Endothelium intact				Endothelium Denuded			
	Abdominal aortae		Thoracic aortae		Abdominal aortae		Thoracic aortae		Abdominal aortae		Thoracic aortae		Abdominal aortae		Thoracic aortae	
	E_max_	pEC_50_	E_max_	pEC_50_	E_max_	pEC_50_	E_max_	pEC_50_	E_max_	pEC_50_	E_max_	pEC_50_	E_max_	pEC_50_	E_max_	pEC_50_
Normal	104.9 ± 3.6	4.6 ± 0.1	102.9 ± 3.9	5.1 ± 0.2	111.2 ± 5.2	5.5 ± 0.2	113.6 ± 5.7	5.2 ± 0.1	113.9 ± 3.1	4.9 ± 0.1	109.9 ± 2.3	5.3 ± 0.1	131.6 ± 1.4	5.5 ± 0.3	120.9 ± 4.7	5.0 ± 0.1
Diabetic	142.9 ± 3.4^a^	5.9 ± 0.4	141.0 ± 4.1^a^	5.7 ± 0.2	157.4 ± 6.1	5.9 ± 0.2	153.7 ± 5.2^a^	5.8 ± 0.3	146.7 ± 4.9^a^	5.5 ± 0.3	158.3 ± 2.3^a^	5.8 ± 0.3	176.3 ± 5.7^a^	5.9 ± 0.3	168.4 ± 5.5^a^	6.0 ± 0.3
Quercetin (diabetic)	126.6 ± 2.0^b^	5.3 ± 0.2	129.4 ± 4.2	5.5 ± 0.2	139.9 ± 3.8	5.7 ± 0.2	134.9 ± 4.5^b^	5.6 ± 0.2	134.9 ± 4.5^b^	5.3 ± 0.2	130.1 ± 3.2^b^	5.6 ± 0.1	150.6 ± 4.4^b^	5.31 ± 0.3	144.7 ± 5.7^b^	6.1 ± 0.9
Metformin (diabetic)	126.1 ± 3.4^b^	5.6 ± 0.3	127.6 ± 4.3	5.3 ± 0.2	130.4 ± 5.5	5.8 ± 0.2	130.8 ± 6.8^b^	5.5 ± 0.2	132.3 ± 6.3^b^	5.44 ± 0.2	127.4 ± 1.7^b^	5.4 ± 0.2	142.9 ± 4.5^b^	5.5 ± 0.3	137.1 ± 6.1^b^	5.7 ± 0.2
Quercetin + Metformin (diabetic)	115.5 ± 5.0^bcd^	5.1 ± 0.1	112.1 ± 4.0^bc^	5.3 ± 0.1	120.5 ± 3.8	5.6 ± 0.2	122.1 ± 4.6^b^	5.4 ± 0.1	125.1 ± 1.9^b^	5.3 ± 0.1	119.7 ± 3.9^b^	5.5 ± 0.1	139.9 ± 6.5^bc^	5.1 ± 0.3	132.0 ± 6.5^b^	5.9 ± 0.2

Data represent mean ± SEM.

The alphabet, ‘a’ shows a significant difference in comparison with normal control (*p* < 0.05).

The alphabet, ‘b’ shows a significant difference in comparison to diabetic control (*p* < 0.05).

The alphabet, ‘c’ shows a significant difference in comparison with a quercetin treatment group (*p* < 0.05).

The alphabet, ‘d’ shows a significant difference in comparison with the metformin treatment group (*p* < 0.05).

**Table 2:** Effect of test compounds on acetylcholine (Ach) induced relaxation in aortae.

Treatment groups	Acetylcholine (Ach) induced relaxation in aortae							
	Aortae with perivascular adipose tissue (PVAT)				Aortae without PVAT			
	Endothelium intact				Endothelium intact			
	Abdominal aortae		Thoracic aortae		Abdominal aortae		Thoracic aortae	
	R_max_	pEC_50_	R_max_	pEC_50_	R_max_	pEC_50_	R_max_	pEC_50_
Normal	104.8 ± 4.2	5.2 ± 0.1	105.2 ± 4.4	5.6 ± 0.1	101.6 ± 0.7	5.4 ± 0.2	104.6 ± 5.0	5.4 ± 0.1
Diabetic	62.9 ± 4.3^a^	3.4 ± 0.2	64.6 ± 3.0^a^	3.6 ± 0.2	64.5 ± 4.5^a^	3.5 ± 0.2	69.2 ± 6.2^a^	3.5 ± 0.2
Quercetin (diabetic)	83.4 ± 2.5^b^	4.8 ± 0.1	84.7 ± 2.2^b^	4.8 ± 0.1	79.3 ± 1.2^ab^	4.5 ± 0.2	81.2 ± 1.9^ab^	4.8 ± 0.2
Metformin (diabetic)	83.0 ± 1.4^b^	4.8 ± 0.1	87.2 ± 4.2^b^	5.2 ± 0.2	84.4 ± 4.7^ab^	4.6 ± 0.2	84.6 ± 2.1^ab^	4.8 ± 0.1
Quercetin + Metformin (diabetic)	96.8 ± 2.2^bcd^	5.0 ± 0.1	95.3 ± 3.8^b^	5.1 ± 0.1	93.8 ± 2.4^b^	5.1 ± 0.1	100.2 ± 2.6^bcd^	5.2 ± 0.1

Data represent mean ± SEM.

The alphabet, ‘a’ shows a significant difference in comparison with normal control (*p* < 0.05).

The alphabet, ‘b’ shows a significant difference in comparison to diabetic control (*p* < 0.05).

The alphabet, ‘c’ shows a significant difference in comparison with a quercetin treatment group (*p* < 0.05).

The alphabet, ‘d’ shows a significant difference in comparison with the metformin treatment group (*p* < 0.05).

**Table 3:** Effect of test compounds on sodium nitroprusside (SNP) induced relaxation in aortae.

Treatment groups	Sodium nitroprusside (SNP) induced relaxation in aortae															
	Aortae with perivascular adipose tissue (PVAT)								Aortae without PVAT							
	Endothelium intact				Endothelium Denuded				Endothelium intact				Endothelium Denuded			
	Abdominal aortae		Thoracic aortae		Abdominal aortae		Thoracic aortae		Abdominal aortae		Thoracic aortae		Abdominal aortae		Thoracic aortae	
	R_max_	pEC^50^	R_max_	pEC_50_	R_max_	pEC_50_	R_max_	pEC_50_	R_max_	pEC_50_	R_max_	pEC_50_	R_max_	pEC_50_	R_max_	pEC_50_
Normal	105.9 ± 2.0	6.16 ± 0.1	103.8 ± 1.1	5.9 ± 0.1	105.5 ± 1.8	5.8 ± 0.1	105.2 ± 1.7	5.3 ± 0.1	108.2 ± 1.7	6.0 ± 0.2	108.5 ± 2.3	6.50 ± 0.1	108.5 ± 1.4	6.3 ± 0.1	112.2 ± 1.8	6.6 ± 0.1
Diabetic	92.6 ± 2.1	5.7 ± 0.2	92.2 ± 0.7	5.3 ± 0.2	90.1 ± 1.4	5.0 ± 0.1	91.5 ± 0.8	5.2 ± 0.1	86.2 ± 1.4	5.4 ± 0.19	92.6 ± 0.6	5.3 ± 0.7	92.2 ± 1.9	5.6 ± 0.3	93.4 ± 0.8a	5.3 ± 0.1
Quercetin (diabetic)	94.9 ± 1.0	6.0 ± 0.1	96.4 ± 0.7	6.0 ± 0.1	99.0 ± 0.4a	5.5 ± 0.0	97.6 ± 0.9	5.4 ± 0.1	97.3 ± 3.5	5.9 ± 0.1	103.7 ± 1.3	5.85 ± 0.1	97.8 ± 0.6	5.9 ± 0.3	98.7 ± 0.5	6.2 ± 0.1
Metformin (diabetic)	96.9 ± 0.5	5.9 ± 0.2	97.2 ± 0.7	5.6 ± 0.1	96.9 ± 0.3	5.6 ± 0.1	96.4 ± 1.0	5.5 ± 0.1	93.9 ± 1.7	6.0 ± 0.1	99.8 ± 0.8	6.4 ± 0.1	101.9 ± 1.1	6.3 ± 0.1	104.8 ± 1.0	6.1 ± 0.1
Quercetin + Metformin (diabetic)	99.3 ± 0.8	6.1 ± 0.2	99.7 ± 1.5	6.0 ± 0.1	103.4 ± 1.0b	6.0 ± 0.1	100.7 ± 1.1	5.6 ± 0.2	98.6 ± 0.9	5.9 ± 0.1	103.0 ± 1.5	6.2 ± 0.1	103.0 ± 1.0	6.2 ± 0.1	108.4 ± 1.2	6.1 ± 0.1

Data represent mean ± SEM.

The alphabet, ‘a’ shows a significant difference in comparison with normal control (*p* < 0.05).

The alphabet, ‘b’ shows a significant difference in comparison to diabetic control (*p* < 0.05).

### Histological analysis

#### Liver

**Figure 4:**
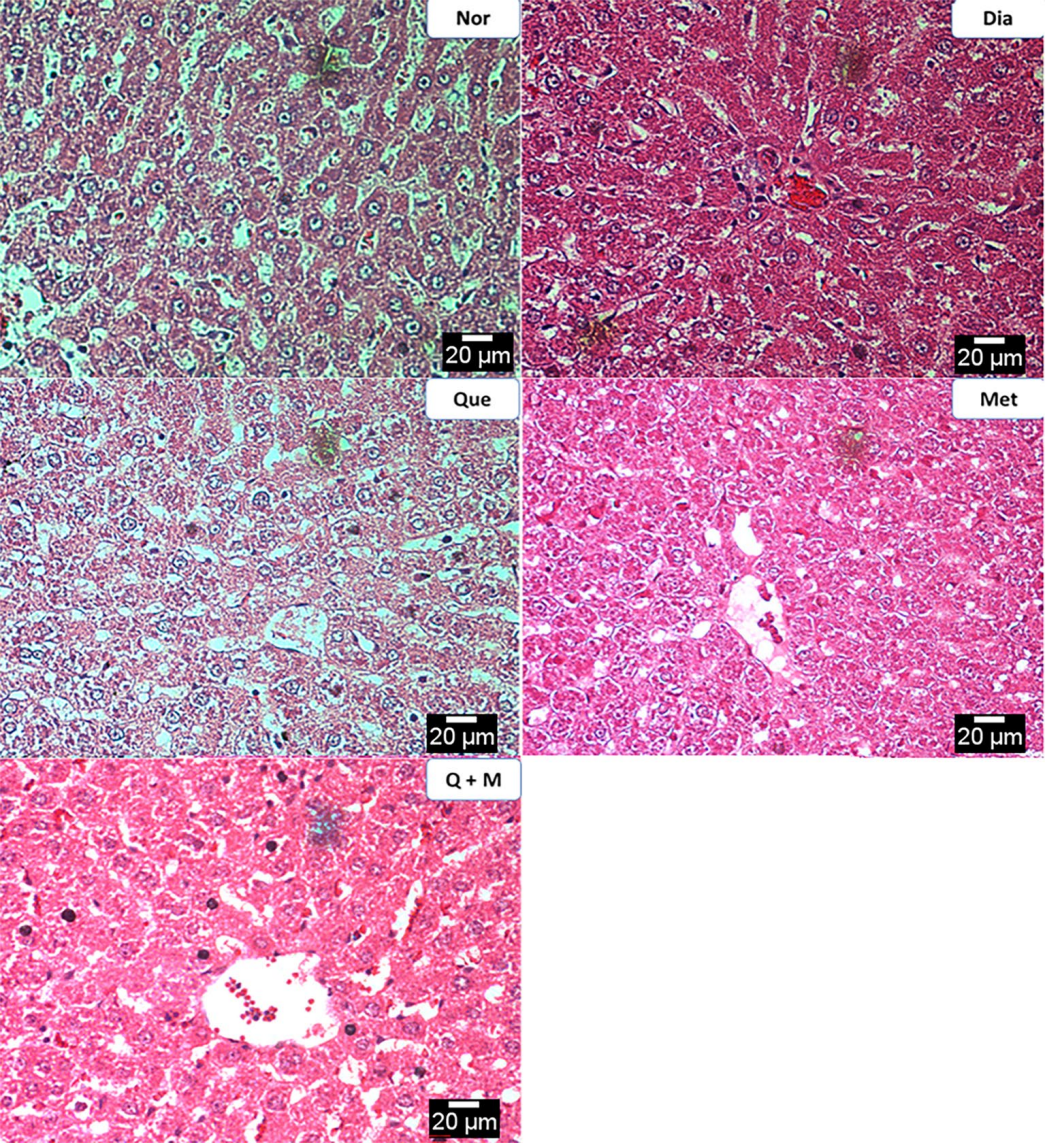
The effect of test compounds on liver histology in diabetic rats. *Nor* Normal, *Dia* Diabetic, *Que* Quercetin treated, *Met* Metformin treated, *Q + M* Quercetin + metformin treated diabetic rats.

Control groups showed mild edematous liver parenchyma with intact architecture and minimal inflammation of periportal areas. Treatment with STZ caused predominantly degenerative changes in the hepatocytes, including fatty, feathery, and hydropic types. The diabetic rats treated with metformin or quercetin showed minimal degenerative changes indicating their protective effect on the hepatocytes. However, the diabetic rats treated with a combination of metformin and quercetin showed very minimal degenerative changes, indicating a better protective effect on the hepatocytes than these drug’s independent effects, as shown in Fig. 4.

#### Kidney

**Figure 5:**
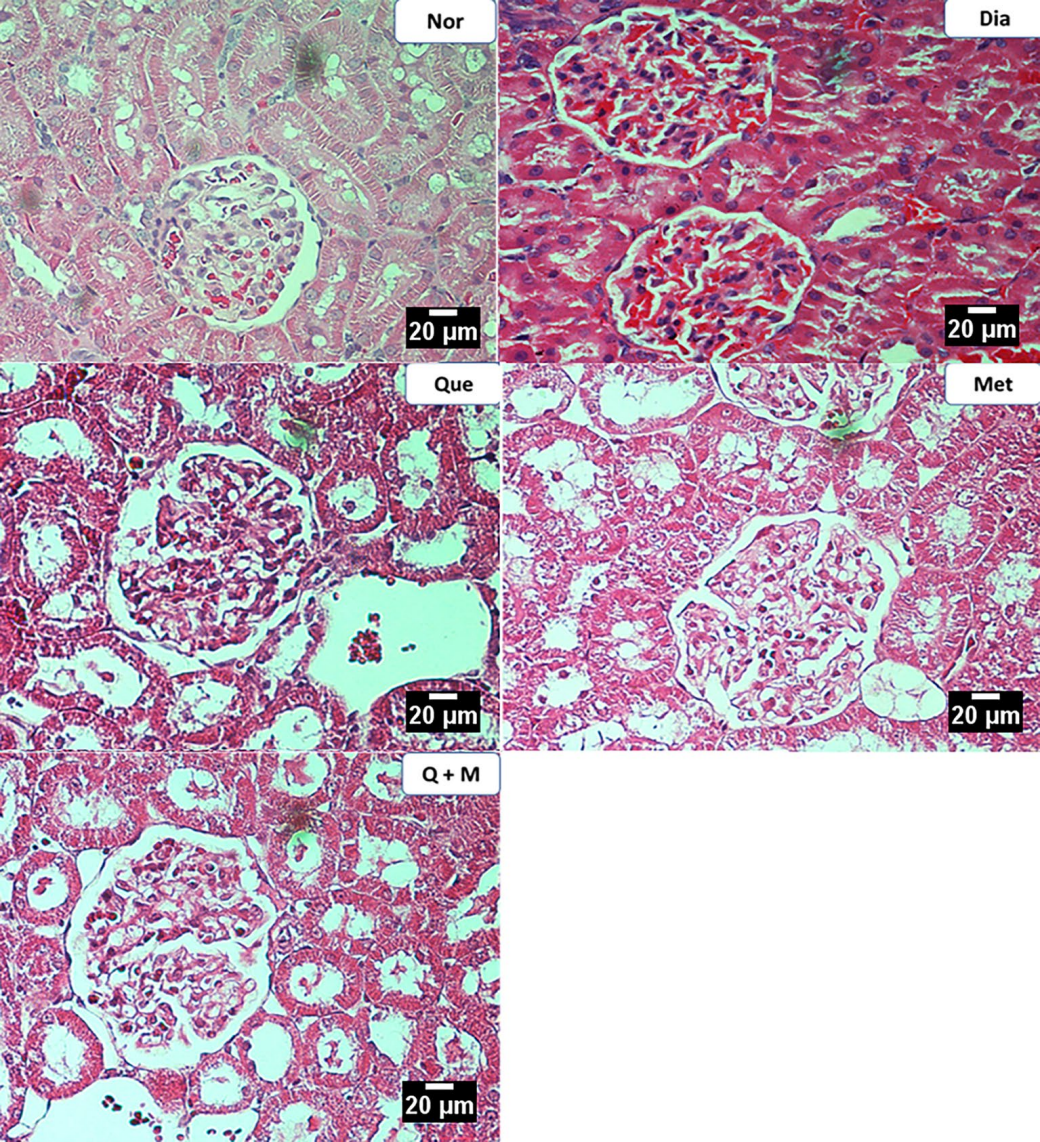
The effect of test compounds on kidney histology in diabetic rats. *Nor* Normal, *Dia* Diabetic, *Que* Quercetin treated, *Met* Metformin treated, *Q + M* Quercetin + metformin treated diabetic rats.

Normal control observed renal parenchyma with mild interstitial oedema near glomeruli. The diabetic rats showed predominant changes in the glomerulus, such as (1) basement membrane thickening, (2) mesangial proliferation, and (3) thickened blood vessels in the interstitium with mild inflammation. The metformin or quercetin treated rats showed similar changes in the diabetic group. However, the diabetic rats treated with the combination of metformin and quercetin showed only very mild glomerular changes indicating a better protective effect on the kidneys (Fig. 5).

#### Pancreas

**Figure 6:**
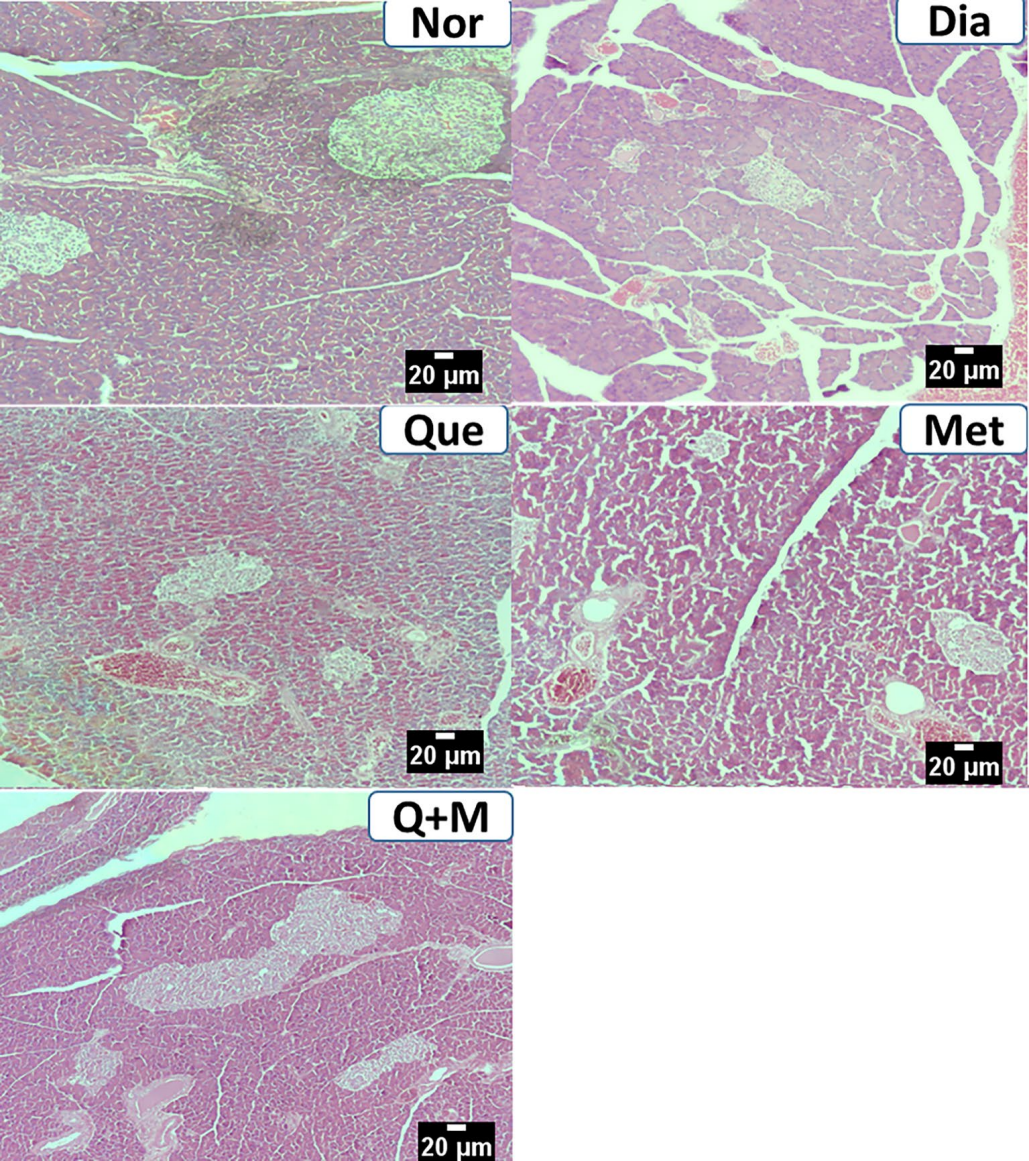
The effect of test compounds on pancreas histology in diabetic rats. *Nor* Normal, *Dia* Diabetic, *Que* Quercetin treated, *Met* Metformin treated, *Q + M* Quercetin + metformin treated diabetic rats.

The rats in the control group showed normal exocrine and endocrine cell proportions. The exocrine acinar cells were arranged in lobules, endocrine islet cells were found embedded inside the lobules of acinar cells and were enclosed by a thin capsule. Treatmenrt with STZ caused a drastic reduction of the volume of islets per lobule and showed focal areas of pale eosinophilic material and a small number of atrophic cells. This eosinophilic material was also seen surrounding the small blood vessels. The diabetic rats treated with metformin or quercetin showed mild degenerative changes. An occasional islet showed lymphocytic insulitis. The group treated with a combination of metformin and quercetin showed islets with several cells but with a lesser volume compared to the control. Only mild infiltration of inflammatory cells and eosinophilic deposits were observed in this group (Fig. 6).

#### Abdominal and thoracic aortae

**Figure 7:**
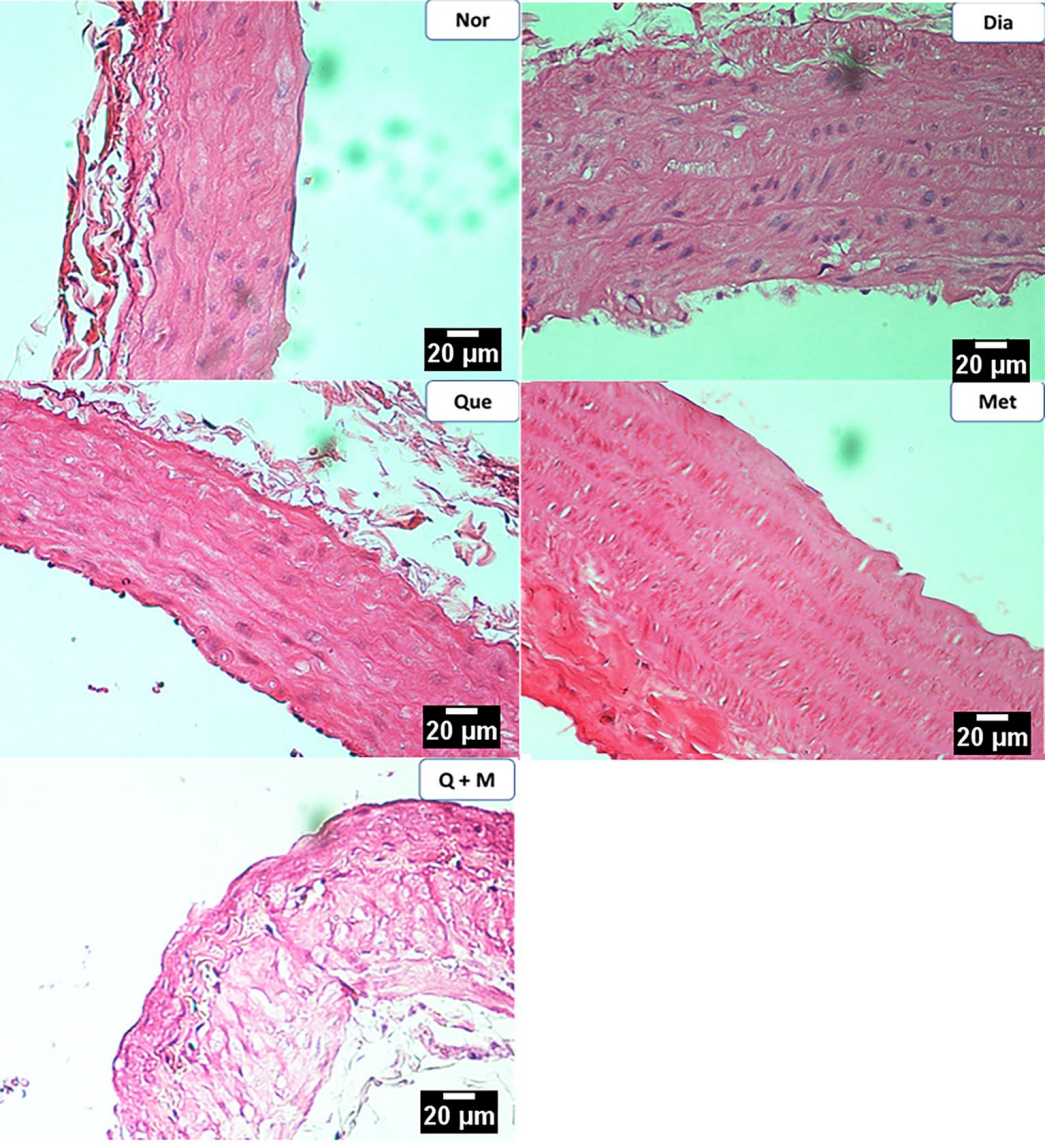
The effect of test compounds on abdominal aortae histology in diabetic rats. *Nor* Normal, *Dia* Diabetic, *Que* Quercetin treated, *Met* Metformin treated, *Q + M* Quercetin + metformin treated diabetic rats.

**Figure 8:**
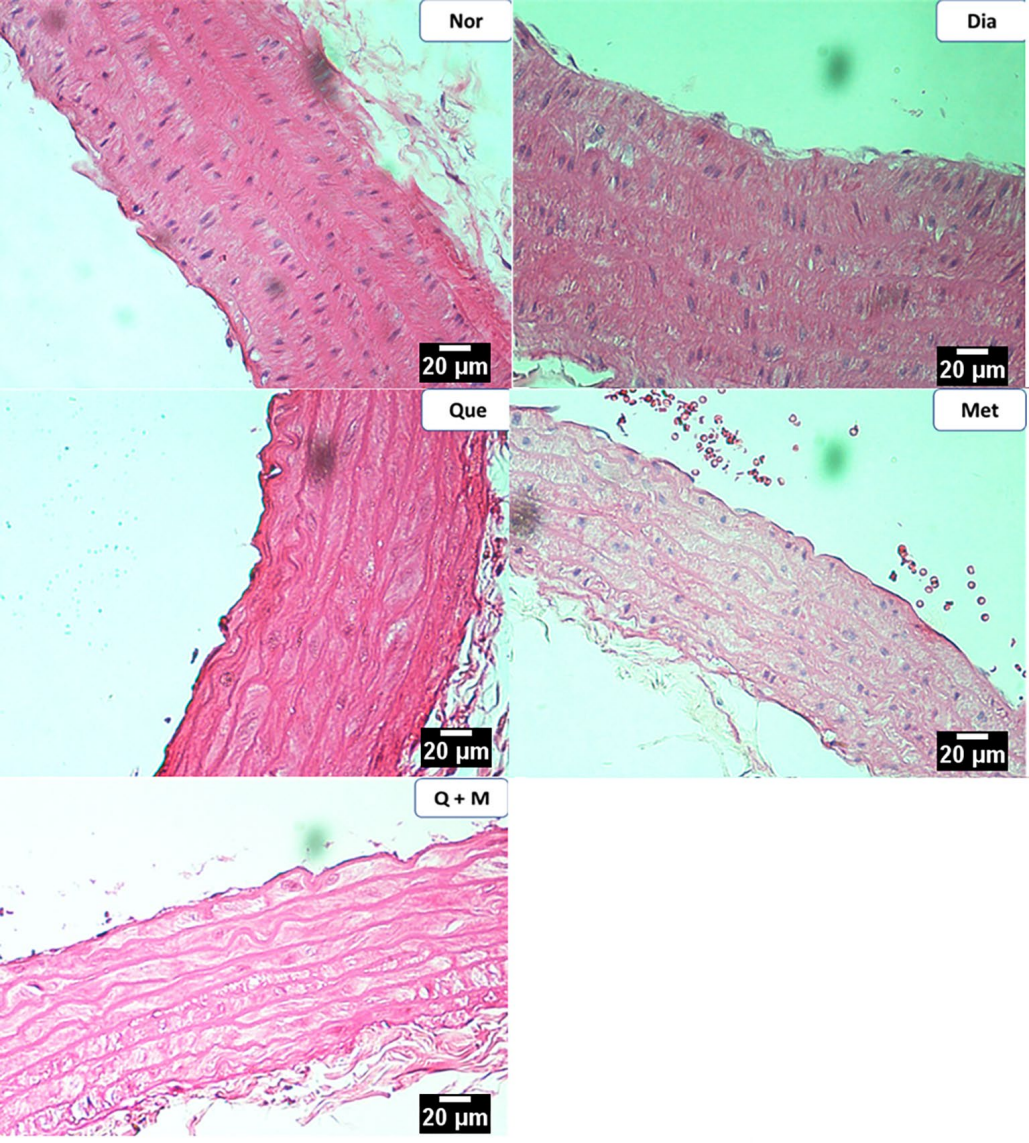
The effect of test compounds on thoracic aortae histology in diabetic rats. *Nor* Normal, *Dia* Diabetic, *Que* Quercetin treated, *Met* Metformin treated, *Q + M* Quercetin + metformin treated diabetic rats.

The rats in the control group showed a typical blood vessel architecture. STZ treatment caused focal fat depositions in tunica intima and mild tunica media enlargement. The diabetic rats treated with metformin or quercetin showed a mild degree of fatty deposits in the tunica intima focally, indicating the protective effect on the arteries. The diabetic rats treated with a combination of metformin and quercetin showed minimal fatty changes indicating a better protective effect on the aortae (Figs. 7, 8).

### Immunohistochemistry

The expression levels of eNOS, 3-nitrotyrosine, VCAM-1, CD31, and SIRT1 in abdominal aortae were comparatively improved with quercetin + metformin combined treatment individual treatments. In diabetic rats, the 3-nitrotyrosine, VCAM-1, and CD31 expression were strong with 3 + density in s80% of the endothelium; however, eNOS and SIRT1 levels were less (between zero to 1 + density) in 10% of the endothelium. The sections of the normal control group showed a 2 + density of the endothelium of eNOS, 1 + in 30–40% of the endothelium of SIRT1, and minimal to 1 + density in 10–40% of endothelium for 3-nitrotyrosine, VCAM-1 and CD31 expression (Fig. S1).

The expression levels of eNOS, 3-nitrotyrosine, VCAM-1, and CD31 observed in thoracic aortae were similar to the levels in observed in the abdominal aortae except that SIRT1 expressed at high density with 3 + in 80% of the endothelium of diabetic rats and lower in normal control (1 + density in 10–40% of endothelium). The combination of quercetin + metformin improved the levels compared to individual treatment (Fig. S2).

## Discussion

Hyperglycemia is the major contributing factor for dyslipidemia and endothelial dysfunction, proceed to cardiovascular complications^18^. Thus, targeting hyperglycemia, dyslipidemia, and endothelial dysfunction is a promising therapeutic approach. Besides this, a combination of drugs and natural products would treat complex diseases. In this milieu, the first-choice antidiabetic drug “metformin” was investigated along with a flavonoid “quercetin” in the treatment of hyperglycemia, dyslipidemia, and endothelial dysfunction in diabetic rat models. As per our knowledge, this is the first report on the synergistic effect of metformin and quercetin in diabetes.

The treatment with quercetin or metformin reduced the plasma glucose levels in diabetic rats. However, the diabetic rats treated with the combination of quercetin and metformin showed the most significant activity in reversing the elevated blood glucose levels compared to the treatment with quercetin or metformin alone. Both quercetin and metformin are well known for lowering plasma glucose levels and improving liver gluconeogenesis and serum lipid parameters^3,19,20^. The antihyperglycemic effect of metformin + quercetin has not been reported so far. The synergistic effect between quercetin and resveratrol in reversing hyperglycemia and endothelial dysfunction were reported^21^.

Dyslipidemia associated with diabetes is an important risk factor for endothelial dysfunction, which leads to atherosclerosis, myocardial infarction and coronary diseases^22^. The present study revealed that the combination of quercetin + metformin is more effective in reversing the lipid levels in diabetic rats than either quercetin or metformin alone. Garimella et al.^23^ showed that metformin treatment improves the lipid profile of individuals with T2DM. Mariee and co-workers showed that quercetin treatment significantly decreased triglycerides, total cholesterol, serum low-density lipoprotein, and high-density lipoprotein levels^24^. However, the effect of quercetin + metformin combination on lipid profile has not been investigated so far.

Quercetin, metformin and their combination reversed the elevated levels of liver enzymes, aspartate aminotransferase (AST), and alanine aminotransferase (ALT) in diabetic rats. Moreover, plasma lactate dehydrogenase (LDH) levels were improved with combination treatment compared with individual treatments and control. The mechanism by which the combination therapy reverses the diabetic complications are not fully understood. Still, it could be due to the restoration of genes involved in glucose/lipid metabolism, liver function, cardiovascular system, and inflammation/immunity, which were altered by STZ-nicotinamide injection. Histological studies supported these results. Quercetin and metformin showed a synergistic effect in protecting hepatocytes and glomeruli.

Endothelium, a permeable barrier between the bloodstream and the outer vascular wall, regulates the vascular tone and structure. Endothelial cells synthesize and secrete nitric oxide (NO) through the action by endothelial NO synthase (eNOS). Under normal conditions, endothelium induces the production and release of NO. The cardiovascular protective effect of NO is mediated by preventing leukocyte adhesion, migration of leukocytes into the arterial wall, proliferation of the muscle cell, platelet adhesion, aggregation, and adhesion molecule expression. In diabetic conditions, the endothelium loses its protective role and becomes a pro-atherosclerotic structure. Perivascular adipose tissue (PVAT) plays a crucial role in regulating vascular functions. PVAT is known to release an unknown PVAT- released relaxation factor (PVAF) in rat aortae and other arteries^25^. In rat aortae, PVAT alters the relaxation responses to blood vessels by NO release and involving hydrogen peroxide production by PVAT. Therefore, PVAT has similar properties as that of the endothelium in regulating vascular tone and functions. In diabetic conditions, PVAT undergoes functional and structural alterations that impact vascular functions^26^. In this study, quercetin, metformin, and the combination increased the ACh-induced endothelium-dependent relaxation, SNP-induced endothelium-independent relaxation and reduced the α1-adrenergic agonist, PE-induced contraction in diabetic rat aortic rings ex vivo but did not have such effect in normal rat aortic rings. ACh and SNP showed reduced responses in aorta relaxation, whereas PE enhanced the aorta contraction in diabetic rats^8^. Increased production of superoxide anions inhibits the endothelial relaxation and enhanced the α1- receptor-induced contractions. These actions are mediated via either enhancing the vascular smooth muscle contraction or decreasing the nitric oxide level^8^.

Quercetin is reported to protect the eNOS levels in hyperglycemic conditions. It has been reported in the literature that metformin improves endothelial function through upregulation of eNOS^15,27–31^. Immunohistochemical studies revealed that eNOS (in abdominal and thoracic aortae) was downregulated in diabetic rats. The treatment of diabetic rats with test compounds (quercetin, metformin, and the combination) upregulated the eNOS levels (abdominal and thoracic aortae) compared to the untreated diabetic control. There was no significant difference in the activity of test compounds on eNOS levels in either abdominal or thoracic aortae.

Nitrotyrosine content is higher in endothelial cells of diabetic patients and thus induces endothelial dysfunction. The increase in NAD(P)H oxidase–induced O_2_ − production in diabetic conditions initiate oxidative/nitrative stress. Inactivation of NO by O_2_ − anion is associated with reduced NO bioavailability and thus causes endothelial dysfunction^32^. Immunohistochemical studies revealed that vascular tissues (abdominal and thoracic aortae) in diabetic control rats contain significantly elevated levels of 3-nitrotyrosine, compared to that in normal control. Nitrotyrosine (in abdominal and thoracic aortae) was highly upregulated in diabetic rats. The treatment with test compounds (quercetin, metformin, and the combination) reversed the elevated nitrotyrosine levels (abdominal and thoracic aortae) in diabetic rats. There was no significant difference in the activity of test compounds on nitrotyrosine levels in abdominal aortae versus thoracic aortae.

Vascular cell adhesion protein-1 (VCAM-1) facilitates the adhesion of lymphocytes, monocytes, basophils, and eosinophils to the surface of the endothelial cells, which promotes migration through the endothelium^33^. In this study, increased oxidative stress was associated with increased VCAM-1 in both abdominal and thoracic aortae. The combination therapy reverses the diabetes induced endothelial dysfunction through downregulation of the expression of VCAM-1 levels (abdominal and thoracic aortae) as compared to that in diabetic control. However, there was no significant difference in the VCAM-1 levels in abdominal versus thoracic aortae.

CD31 is a member of the immunoglobulin family, designated as PECAM-1 (platelet endothelial cell adhesion molecule 1). It is present in the endothelial intercellular junction. CD31 facilitates adhesion between endothelial cells (EC) and the inflammatory cells during inflammatory conditions and angiogenesis^34–36^. It has been reported that an increase in the CD31 number is associated with cardiovascular risk^35^. The treatment of diabetic rats with test compounds (quercetin, metformin, and combination) downregulated the CD31 levels in the abdominal and thoracic aortae.

Hyperglycemia in diabetes decreases SIRT-1 expression and thus activates p53 by increasing its acetylation. It has been reported that activation of SIRT-1 prevents hyperglycemia-induced vascular cell senescence and thereby improves vascular function in diabetic conditions^37^. In addition, the reductions in SIRT-1 deacetylation of eNOS can impair endothelium-dependent dilation (EDD)^38^. Quercetin, in diabetes, activates SIRT-1 leading to eNOS phosphorylation, NO production and intracellular cGMP in endothelial progenitor cells. It is reported that quercetin upregulates SIRT-1. Metformin has been shown to improve endothelial function and protect the macro-and microvasculature in diabetes^14,39^. It has been suggested that SIRT-1 is a key target for the endothelial-protective action of metformin. In this study, the treatment in diabetic rats with test compounds (quercetin, metformin, and combination) and the SIRT-1 levels (abdominal and thoracic aortae) in diabetic rats were found to be upregulated compared to those treated to diabetic control. There was no significant difference in the activity of test compounds on SIRT-1 levels in either abdominal or thoracic aortae.

In conclusion, the combination of quercetin with metformin showed a greater antidiabetic effect compared with metformin or quercetin individually. In addition, the combination effectively reversed the hyperglycemia-induced endothelial dysfunction in diabetic rats and regulated the expression of eNOS, 3-nitrotyrosine, VCAM-1, CD31 and SIRT-1 markers. Overall, the results from this study suggest that quercetin potentiates the activity of metformin to control diabetes.

## Methods

### Animals

Male Sprague Dawley rats (8 weeks old, 180 –220 g weight) were purchased from Animal Resource Unit, Faculty of Veterinary Medicine, University Putra Malaysia and housed 7 days for acclimatization (24 ± 1 °C, 50 ± 5% humidity and with a 12 h light/12 h dark cycle) before the experiments. All the rats had free access to standard rat chow (Gold Coin Feedmills, Malaysia) and reverse osmosis water. The experimental protocol was approved (Approval No.: PhD 4.11/JCM-46/2011) by the Research & Ethics Committee, International Medical University, Malaysia. All methods involving rats were carried out in accordance with standard conventional guidelines and regulations. We have also conducted the experiments in accordance with ARRIVE guidelines. Experimental animals were euthanized by cervical dislocation method which is one of the common methods for euthanasia.

### Experimental design and induction of type 2 diabetes

Seventy-five rats were divided into five groups (15 rats in each group). The group of rats fed with a standard diet was referred to as normal control. The other rats were fasted 6–8 h prior to inducing diabetes. The diabetes was induced by intraperitoneal injection of streptozotocin (STZ; 60 mg/kg body weight, Amresco, USA) followed by nicotinamide (120 mg/kg body weight, Sigma-Aldrich, Malaysia) in a 15-min interval. On the 7th day after the injection, the plasma glucose levels in rats were greater than 11.0 mmol/l and were confirmed, diabetic. The rats were maintained in this condition for 7 to 8 weeks. Then, the rats were treated for 30 days with quercetin, Metformin, and quercetin + Metformin. The plasma glucose levels in all rats were determined at the baseline and the end of the treatment period.

### Treatment with quercetin, metformin, and quercetin + metformin

Quercetin and metformin solutions were prepared in 1% (w/v) carboxymethyl cellulose (Sigma-Aldrich, Malaysia) and given orally once a day to the type 2 diabetes rats for 30 days. The rats in (1) quercetin group received quercetin 10 mg/kg body weight, (2) metformin group received 180 mg metformin per kg body weight, (3) quercetin + metformin group received 10 mg quercetin followed by 180 mg metformin per kg body weight, and (4) diabetic control received 1% (w/v) carboxymethyl cellulose. The dose of quercetin (10 mg kg^−1^ of body weight), was fixed based on our previous study that was conducted in our laboratory^40^. Similarly, the dose of metformin (180 mg metformin per kg body weight) was decided based on previously published studies that were conducted on rats under similar conditions^41^. Moreover, as the optimal response dose has been already established, we had decided to employ a single dose of quercetin and metformin based on previous reported studies.

### Oral glucose tolerance test

After 30 days of treatment, the diabetic rats were fasted for 12 h and given an oral glucose load of 3 g/kg body weight through gastric intubation. The blood glucose levels were checked at intervals of 30 min up to 120 min by using the Accu-chek Performa blood glucose test strips (Roche Diagnostics, Germany).

### Biochemical analysis

Blood was collected through the retro-orbital sinus of rats (after 30-day treatment), and the plasma was separated following the routine procedure. The plasma was analyzed for (1) aspartate aminotransferase (AST), (2) alanine aminotransferase (ALT), (3) lactate dehydrogenase (LDH), (4) glucose, (5) creatinine (CR), (6) total cholesterol (TC), (7) high-density lipoprotein (HDL), (8) low-density lipoprotein (LDL), and (9) triglycerides (TG) using Siemens Dimension Xpand Plus (Faculty of Veterinary Medicine, University Putra Malaysia).

### Ex-vivo studies with rat aortic rings

The rats were euthanized by cervical dislocation method which is one of the common methods for euthanasia. The descending thoracic and abdominal aortae rings of the rats (~ 3–5 mm) were carefully removed as described by Boutouyrie et al.^42^. The aortae associated perivascular adipose tissue (PVAT) and endothelium (ED) were removed and categorized as indicated below. PVAT + ED: aortae rings with undamaged PVAT and intact ED. PVAT ˗ ED: aortae rings with undamaged PVAT and no ED. ˗ PVAT + ED: aortae rings with intact ED and no PVAT. ˗ PVAT ˗ ED: aortae rings without ED and PVAT. The isometric tension, effect of phenylephrine, acetylcholine and sodium nitroprusside on aortic rings were investigated.

### Measurement of isometric tension

The inner glass-jacketed organ bath vessel was filled with 7 ml of Krebs physiological salt solution (KPSS (g/l); NaCl: 6.96; NaHCO_3_: 2.10; MgSO_4_: 0.29; KCl: 0.35; KH_2_PO_4_: 0.14; CaCl_2_: 0.350; glucose: 2.0; EDTA: 0.022) and bubbled continuously with 95% oxygen and 5% carbon dioxide at 37 °C. The aortic rings were then hooked on to an isometric force–displacement transducer (AD Instruments, Australia) and left in the Krebs solution at a tension of 1.5 g for 90 min for equilibrating and stabilizing the aortic rings. The aortic response output from the transducer was amplified and recorded in gram tension throughout the experiment using the Power Lab recording system (AD Instruments, Australia). After the equilibration, aortic rings were immersed in potassium chloride solution (80 mM) for 5 min to fix a baseline contractile response. The percentage of the KCl contraction was used to express the contractile response to phenylephrine. This step was repeated 3 times over intervals of 5 min. Then, the tissue was challenged with phenylephrine (10 μM), and the contraction was observed until it reached a plateau. Following this, acetylcholine (10 μM) was added, and the relaxant response was observed. The endothelium is considered intact if the percentage ACh-induced relaxation was higher than 50% of the initial phenylephrine-induced contraction. If the relaxation induced by acetylcholine was less than 50%, the endothelium was considered to be denuded.

### Effect of phenylephrine, acetylcholine, and sodium nitroprusside on aortic rings

After establishing the endothelium integrity, the tissues were treated with increasing concentrations of phenylephrine (1 nM to 1 µM) at intervals of 4 min between contractions that lasted for 5 min. After the test, the tissues were washed several times using Krebs solution until the tension reached the basal levels in about 30 min. This test was performed to investigate the maximum contraction of tissue.

Simultaneously, to determine the maximum endothelial-dependent relaxation in rat aortae, the tissues were pre-contracted with phenylephrine (10 µM) before being exposed to cumulative acetylcholine concentrations (1 nM to 1 µM).

Besides this, after washing with Krebs solution, the tissues were subjected to pre-contractions with a similar concentration of phenylephrine before they were exposed to cumulative concentrations of sodium nitroprusside (1 nM to 1 µM). This process establishes the maximum endothelial-independent relaxation in the rat's aortae.

### Histological analysis

After the 30 days treatment, the pancreas, liver, kidneys, and aortae were removed from the sacrificed rats. They were cleaned for adipose and connective tissues and fixed in 10% neutral buffered formalin. After fixation, the organs were embedded in paraffin to be cut into thin sections of ~ 4 μm. The appropriate sections were fixed on the glass slides and stained with hematoxylin and eosin. The stained slides were examined under a light microscope.

### Immunohistochemistry

The tissue sections were mounted on glass slides and treated serially with xylene and ethanol (decreasing gradient). After water and 1 × amplifying immunohistochemistry (ICH) buffer wash, the slides were submerged in 1 × epitope unmasking solution and autoclaved. The autoclaved slides were cooled and washed with 1 × ICH buffer for 5 min with rocking. The wet tissue on slides was covered with 1–3 drops of peroxidase block and incubated at 28 °C for 30 min. Then, the tissue slides were washed with 1 × ICH buffer and kept in antibody amplifier™ and amplifying antibody dilution buffer (~ 3 ml) overnight at 4 °C. The tissue slides were washed with 1 × ICH buffer, covered with 1–3 drops of labelled polymer, and incubated at 28 °C for 30 min. The tissue sections were repeatedly covered with labelled polymer during incubation to avoid drying. After incubation, the slides were washed and stained with diaminobenzidine (DAB) solution and counter stained using 1% (w/v) hematoxylin. The stained slides were examined under a light microscope. The following monoclonal antibodies (Abcam, Malaysia) were used in this experiment. eNOS (ab50010; 1:100 dilution), nitrotyrosine (ab10282; 1:100 dilution), VCAM-1 (ab134047; 1:500 dilution), CD-31 (ab119339; 1:100 dilution), and SIRT-1 (ab110304; 1:200 dilution).

### Statistical analysis

All results are stated as mean ± standard error of the mean (SEM). The phenylephrine dose–response curves were plotted as a percentage of high K^+^ solution-induced contraction per mg tissue weight against the phenylephrine concentration (log scale). The acetylcholine dose–response curves were plotted as a percentage of phenylephrine (0.1 µM) contraction per mg tissue weight against acetyl concentration (log scale). The sodium nitroprusside dose–response curves were plotted as a percentage of phenylephrine (0.1 µM) contraction per mg tissue weight against sodium nitroprusside concentration (log scale). The graphs were plotted using non-linear regression. Two-way ANOVA test followed by Bonferroni post-test were used to analyze the statistical significance for differences in responses among the different groups. A significant difference was accepted when the p-value was less than 0.05. All statistical analysis were performed using GraphPad Prism software (version 5; GraphPad Software Inc., USA).

## Supplementary Information

Supplementary Legends.

Supplementary Figure S1.

Supplementary Figure S1.

Supplementary Figure S1.

Supplementary Figure S1.

Supplementary Figure S1.

Supplementary Figure S2.

Supplementary Figure S2.

Supplementary Figure S2.

Supplementary Figure S2.

Supplementary Figure S2.

## Data Availability

The datasets generated during and/or analysed during the current study are available from the corresponding author on reasonable request.
