# Supplementary material for: Quercetin and metformin synergistically reverse endothelial dysfunction in the isolated aorta of streptozotocin-nicotinamide- induced diabetic rats
Source: Sci Rep. 2022 Dec 10;12:21393. doi: 10.1038/s41598-022-25739-5 (PMC9741611; doi:10.1038/s41598-022-25739-5)
Supplement: Supplementary file 1 — Supplementary Legends. [file 41598_2022_25739_MOESM1_ESM.docx]

**Electronic supplementary material**

**Figure S 1** The effect of test compounds on, (a) eNOS, (b) 3-nitrotyrosine, (c) VCAM1-1, (d) CD31, and (e) SIRT1 on T2DM rat’s abdominal aortae. Normal (Nor), Diabetic (Dia), Quercetin treated (Que), Metformin treated (Met), Quercetin + metformin treated (Q + M) diabetic rats.

**Figure S 2** The effect of test compounds on, (a) eNOS, (b) 3-nitrotyrosine, (c) VCAM1-1, (d) CD31, and (e) SIRT1 on T2DM rat’s thoracic aortae. Normal (Nor), Diabetic (Dia), Quercetin treated (Que), Metformin treated (Met), Quercetin + metformin treated (Q + M) diabetic rats.
