# Supplementary figures and images for: Quercetin and metformin synergistically reverse endothelial dysfunction in the isolated aorta of streptozotocin-nicotinamide- induced diabetic rats
Source: Sci Rep. 2022 Dec 10;12:21393. doi: 10.1038/s41598-022-25739-5 (PMC9741611; doi:10.1038/s41598-022-25739-5)

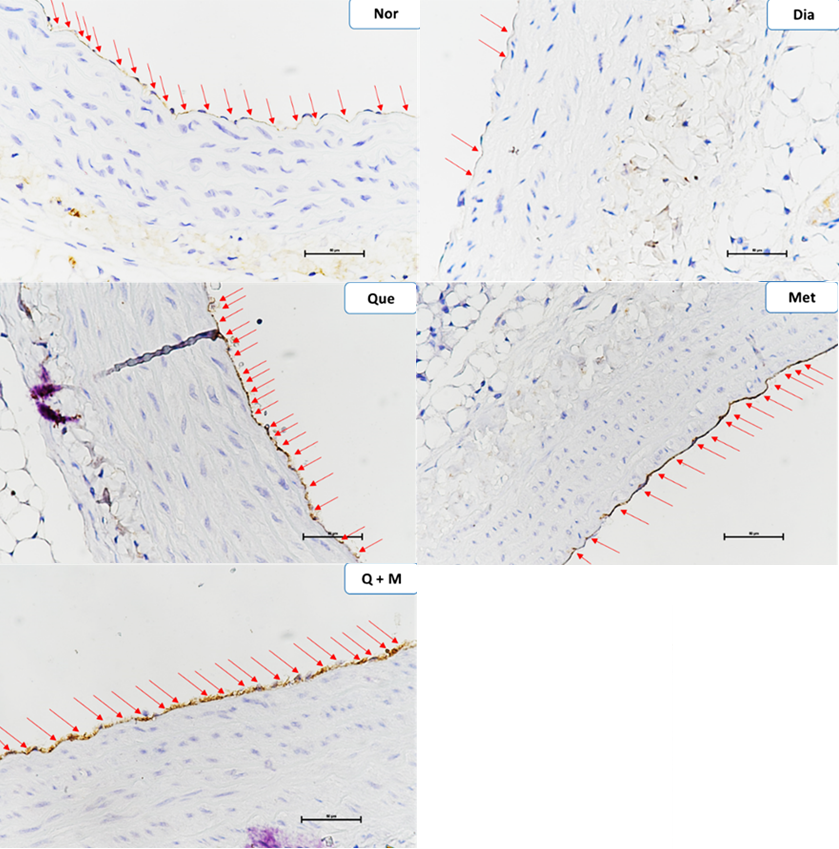

Supplement: Supplementary file 2 — Supplementary Figure S1. [file 41598_2022_25739_MOESM2_ESM.tif]

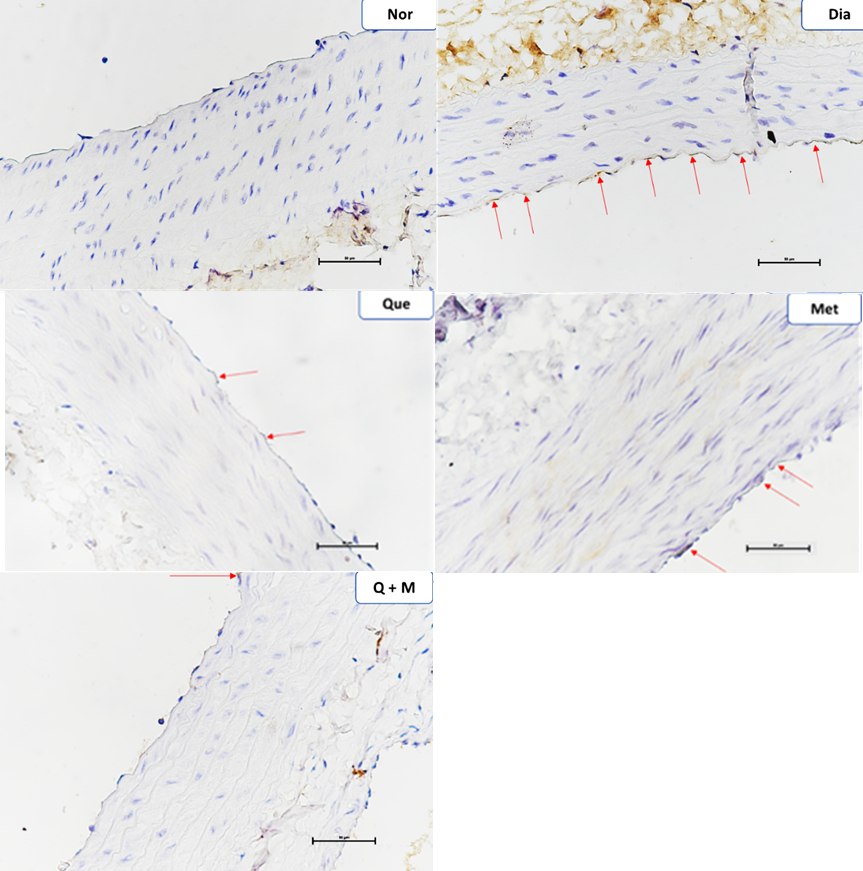

Supplement: Supplementary file 3 — Supplementary Figure S1. [file 41598_2022_25739_MOESM3_ESM.tif]

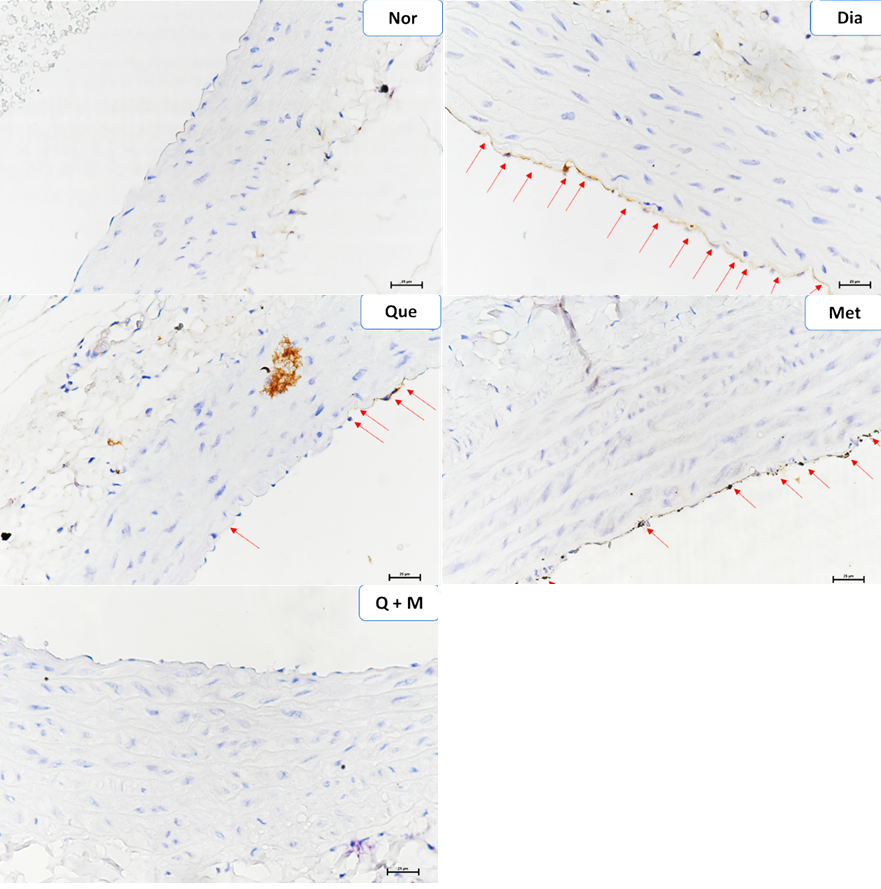

Supplement: Supplementary file 4 — Supplementary Figure S1. [file 41598_2022_25739_MOESM4_ESM.tif]

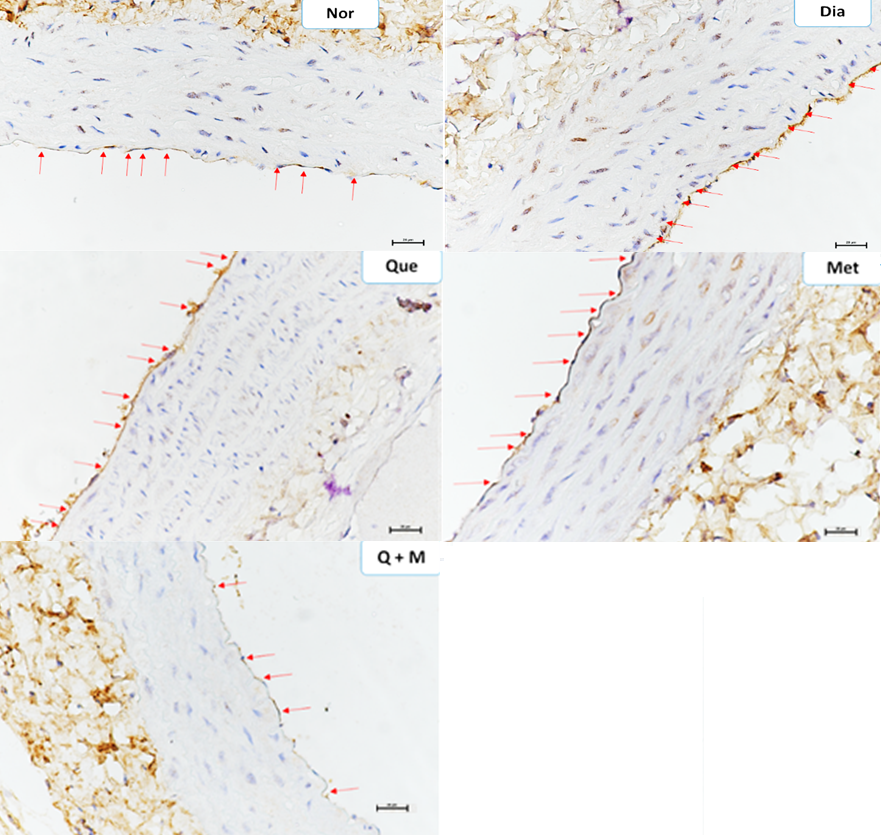

Supplement: Supplementary file 5 — Supplementary Figure S1. [file 41598_2022_25739_MOESM5_ESM.tif]

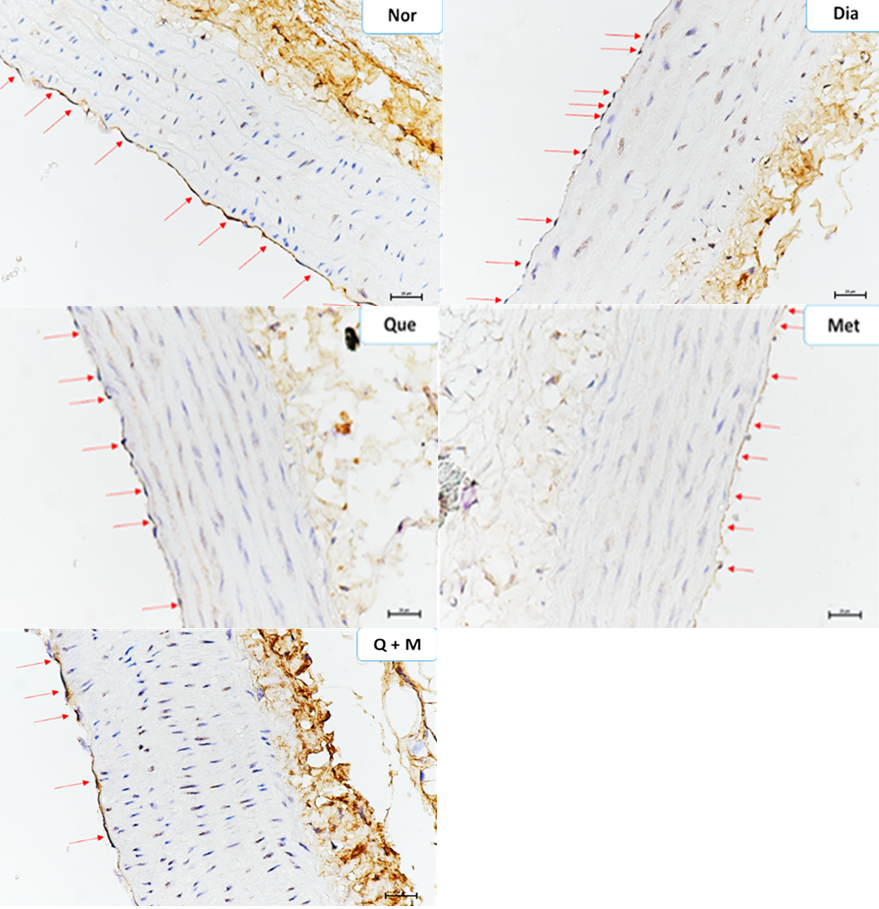

Supplement: Supplementary file 6 — Supplementary Figure S1. [file 41598_2022_25739_MOESM6_ESM.tif]

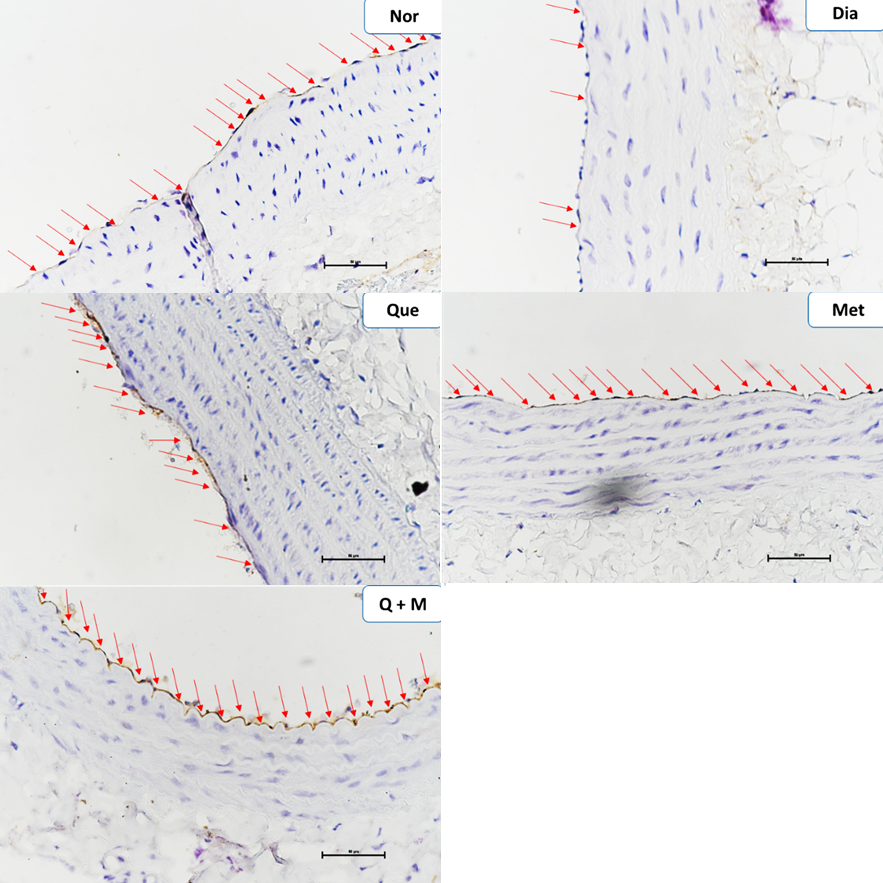

Supplement: Supplementary file 7 — Supplementary Figure S2. [file 41598_2022_25739_MOESM7_ESM.tif]

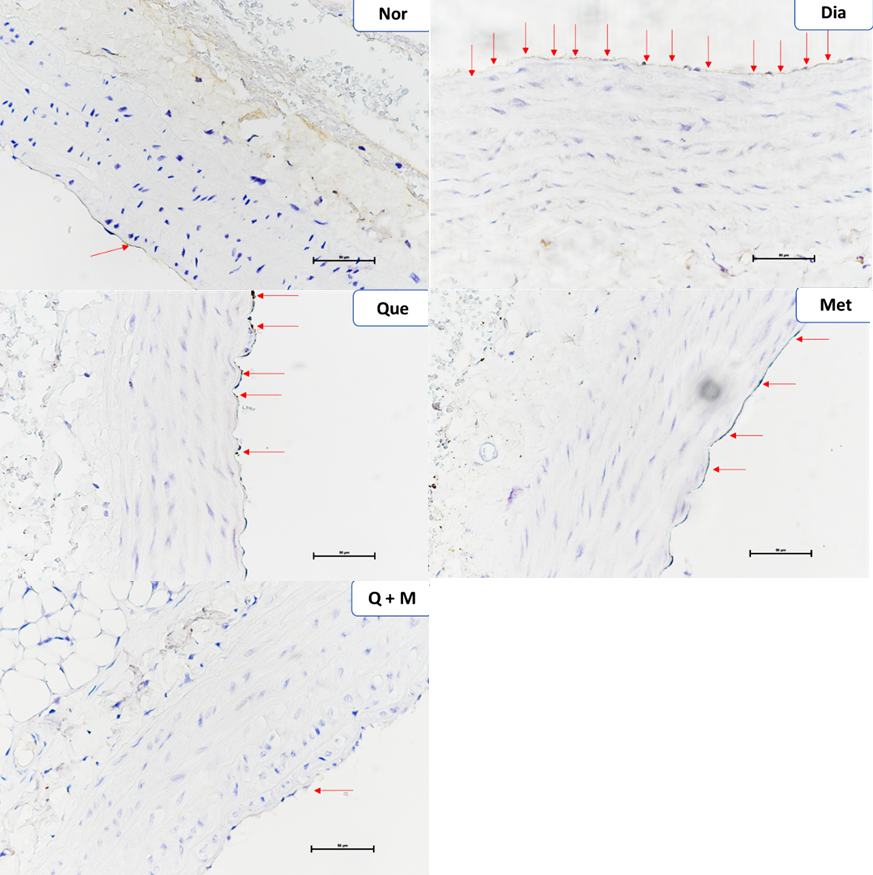

Supplement: Supplementary file 8 — Supplementary Figure S2. [file 41598_2022_25739_MOESM8_ESM.tif]

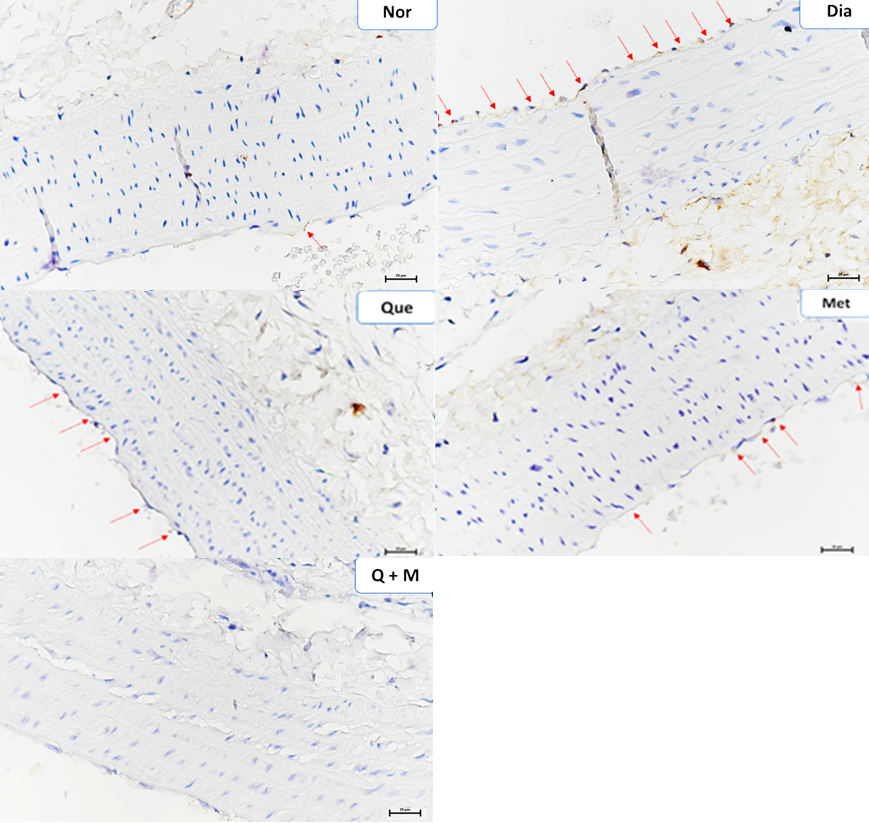

Supplement: Supplementary file 9 — Supplementary Figure S2. [file 41598_2022_25739_MOESM9_ESM.tif]

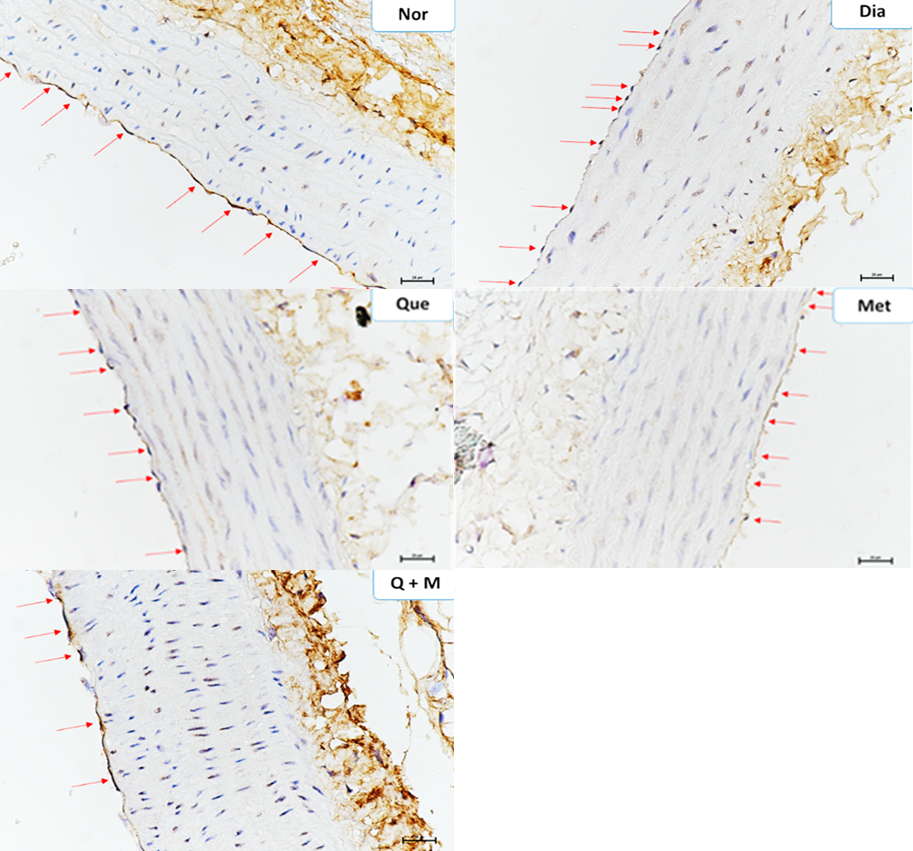

Supplement: Supplementary file 10 — Supplementary Figure S2. [file 41598_2022_25739_MOESM10_ESM.tif]

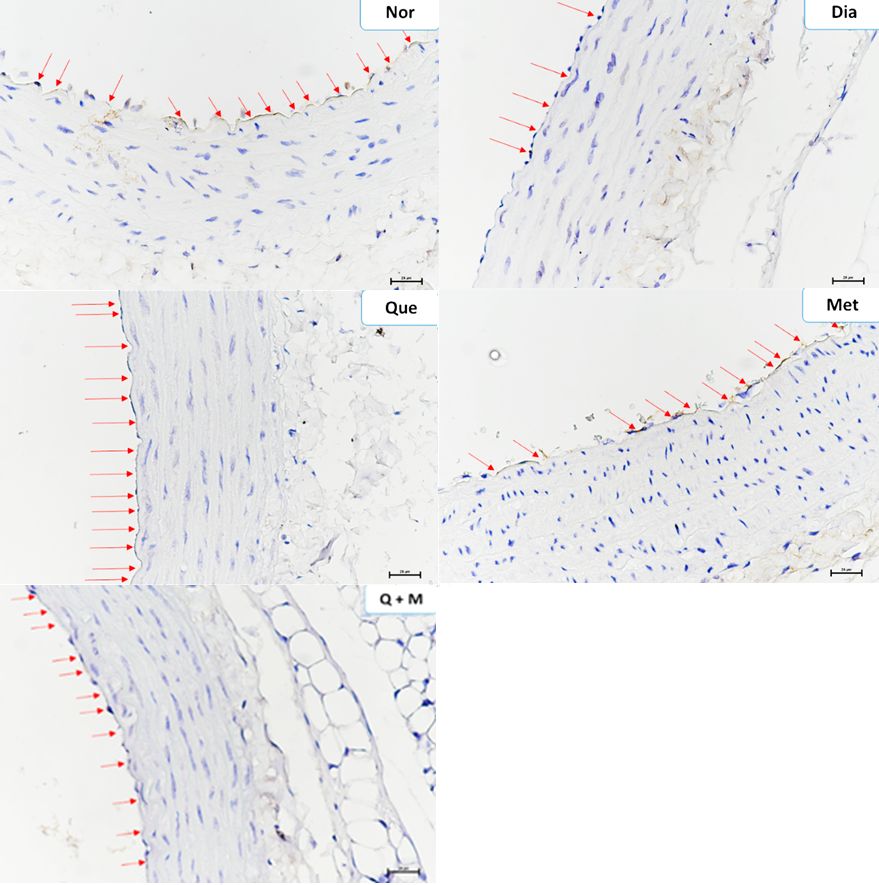

Supplement: Supplementary file 11 — Supplementary Figure S2. [file 41598_2022_25739_MOESM11_ESM.tif]
